# Increased expression of TROP2 in airway basal cells potentially contributes to airway remodeling in chronic obstructive pulmonary disease

**DOI:** 10.1186/s12931-016-0463-z

**Published:** 2016-11-25

**Authors:** Qixiao Liu, Haijun Li, Qin Wang, Yuke Zhang, Wei Wang, Shuang Dou, Wei Xiao

**Affiliations:** 1 Department of Respiratory Medicine, Qilu Hospital, Shandong University, 107 Wenhua West Road, Jinan, China; 2 Department of Cadre Health Care, Qilu Hospital, Shandong University, 107 Wenhua West Road, Jinan, China; 3 Department of Anesthesiology, Qilu Hospital, Shandong University, 107 Wenhua West Road, Jinan, China; 4 Department of Cadre Health Care, Qianfoshan Hospital, 16766 Jingshi Road, Jinan, China

**Keywords:** Airway remodeling, Basal cells, Chronic obstructive pulmonary disease (COPD), Cigarette smoke, Trophoblast cell surface antigen 2 (TROP2)

## Abstract

**Background:**

The airway epithelium of chronic obstructive pulmonary disease (COPD) patients undergoes aberrant repair and remodeling after repetitive injury following exposure to environmental factors. Abnormal airway regeneration observed in COPD is thought to originate in the stem/progenitor cells of the airway epithelium, the basal cells (BCs). However, the molecular mechanisms underlying these changes remain unknown. Here, trophoblast cell surface antigen 2 (TROP2), a protein implicated in the regulation of stem cell activity, was examined in lung tissue samples from COPD patients.

**Methods:**

The expression of TROP2 and hyperplasia index Ki67 was assessed in lung epithelium specimens from non-smokers (*n* = 24), smokers (*n* = 24) and smokers with COPD (*n* = 24). Primary airway BCs were isolated by bronchoscopy from healthy individuals and COPD patients and subsequently transfected with pcDNA3.1-TROP2 or siRNA sequence in vitro. The functional consequences of TROP2 overexpression in BCs were explored.

**Results:**

Immunohistochemistry and immunofluorescence revealed increased TROP2 expression in airway BCs in smokers with COPD compared to nonsmokers and smokers without COPD, and staining was highly localized to hyperplastic regions containing Ki67 positive cells. TROP2 expression was also inversely correlated with airflow limitation in patients with COPD (r = −0.53, *P* < 0.01). pcDNA3.1-TROP2-BCs in vitro exhibited improved proliferation with activation of ERK1/2 phosphorylation signaling pathway. In parallel, changes in vimentin and E-cadherin in pcDNA3.1-TROP2-BCs were consistent with an epithelial-mesenchymal transition (EMT)-like change, and secretion of inflammatory factors IL-1β, IL-8 and IL-6 was increased. Moreover, down-regulation of TROP2 by siRNA significantly attenuated the proliferation of BCs derived from COPD patients. EMT-like features and cytokine levels of COPD basal cells were also weakened following the down-regulation of TROP2.

**Conclusion:**

The results indicate that TROP2 may play a crucial role in COPD by affecting BC function and thus airway remodeling through increased BC hyperplasia, EMT-like change, and introduction of inflammatory molecules into the microenvironment.

## Background

Chronic obstructive pulmonary disease (COPD) is a progressive degenerative lung disease and currently the fourth leading cause of death worldwide [1]. Cigarette smoke and other toxic particles are major risk factors for the development of COPD. A critical feature of COPD is airway remodeling, which is characterized by aberrant repair of the epithelium and accumulation of fibroblasts [2, 3]. As airway epithelium is the primary target of inhaled harmful particles, abnormal tissue repair has become a primary focus in understanding the process of airway remodeling [4].

The airway epithelium consists of four types of cells, including basal cells (BCs), ciliated cells, secretory cells and neuroendocrine cells [5]. BCs include multipotent stem/progenitor progenitor cells of bronchial airway epithelium and make a major contribution to the regeneration of bronchial epithelium [6, 7]. These cells play a key role in the maintenance of the normal airway epithelial architecture through their capacity to self-renew, differentiate into ciliated and secretory cells, and establish interactions with mesenchymal cells [8–11]. Abnormalities in the number and function of BCs may therefore severely affect the regeneration of injured airway epithelium such as in smoking. Basal cell hyperplasia caused by smoking has occurred long before lung function declines, indicating that the development of COPD may begin with these cells [12]. However, little is known about the molecular mechanisms underlying the abnormal biological behavior of BCs in COPD.

Trophoblast cell surface antigen 2 (TROP2), is a type I transmembrane glycoprotein with low to no expression in normal tissues. It provides crucial signals for cells with requirements for self-renewal, survival, and invasion [13, 14]. Although TROP2 has been reported to be highly expressed in various types of epithelial cancers, including colorectal cancer, pancreatic cancer, and oral squamous-cell carcinoma [15–18], TROP2 expression has also been found in stem cells in various tissue types. In human and mouse prostate, the TROP2 expressing subpopulation of BCs possess stem cell capacities such as self-renewal, regeneration and differentiation [19, 20]. Undifferentiated oval cells express TROP2 shortly after activation due to liver injury [21]. TROP2 is also enriched in endometrial-regenerating cells in a dissociated cell tissue recombination assay [22].

These results indicate that TROP2 might play an important role more generally in the regulation of the growth and regeneration of stem cells as well as serve as a molecular marker of them in various adult tissues. Here, protein expression of TROP2 was specifically examined in the development of COPD in smokers. TROP2 was furthermore overexpressed in normal airway BCs in vitro in order to identify potential biological and molecular pathways regulated by the protein. Our results demonstrate that increased expression of TROP2 occurs in the BC compartment of lung tissue samples, indicating that the protein might play a role in aberrant airway repair and remodeling that are characteristic of COPD.

## Methods

### Ethics statement

Protocols for recruitment of patients and healthy, nonsmoking individuals as well as the collection of tissue samples and the analysis of patient data were approved by the ethics committee at Qilu Hospital of Shandong University (Jinan, Shandong, China). Written informed consent was obtained from each individual for participation in the study.

### Patients

Lung tissue samples were obtained from patients (*n* = 72) at Qilu Hospital of Shandong University (Jinan, Shandong, China) during lobectomy or pneumonectomy performed for medical reasons, including lung tumor, pneumatocele and pulmonary cyst. No patient had received corticosteroids (oral or inhaled) within 1 month before tissue collection. The cohort consisted of smokers with COPD (*n* = 24), smokers without COPD (*n* = 24), and nonsmokers (*n* = 24) as controls. The diagnosis of COPD was made according to the guidelines of the Global Initiative for Chronic Obstructive Lung Disease (GOLD) [1]. Tissue samples of ~15 - 25 mm in size (*n* = 2) were removed from the subpleural parenchyma of the lobe at a distance of ≥ 5 cm from the margins of the diseased areas in each patient. The inclusion criterium for smokers with and without COPD was a smoking index of > 10 pack-years which was calculated by multiplying the number of cigarette packs consumed per day (20 cigarettes per pack) with the number of years of smoking. Exclusion criteria were atopic diseases, allergic rhinitis, asthma, diabetes, acute or chronic infections.

### Immunohistochemistry and immunofluorescence

Serial sections (4 μm) were cut from paraffin embedded lung samples, and antigen retrieval was carried out for both immunohistochemistry and immunofluorescence by steaming sections for 15 min in Citrate buffer (pH 6.0). Immunostaining and visualization was performed with the Biotin-Streptavidin HRP Detection Systems (SP) and a DAB color development kit (ZhongShan Golden Bridge Biotechnology, Beijing, China), and the primary antibodies used were TROP2 (5 μg/ml; RD Biosciences, Temecula, NJ, USA) and Ki67 (1:100; Abcam, Cambridge, MA, UK). The mean staining density of TROP2 in airway epithelium was analyzed using Image-Pro Plus 6.0 software. For immunofluorescence, sections were incubated overnight at 4 °C with mixed primary antibodies, including anti-TROP2 (10 μg/ml) and anti-cytokeratin 5 (1:200; Abcam) as a marker for basal cells. For visualization, fluorescently-labeled secondary antibodies were incubated with slides, and images were captured with a Laser confocal microscope (Olympus DP70 CCD camera, Tokyo, Japan). TROP2 was expressed as mean staining density in the airway epithelium. Ki67 index was calculated as the percentage of Ki67 positively stained cell compared with the airway epithelial cells. The area of the airway epithelium and the number of epithelial cells were evaluated using Image-Pro Plus 6.0 software (Media Cybernetics, Silver Spring, MD, USA). The analysis of all slides was performed by an experienced lung pathologist.

### Isolation and culture of BCs

The primary epithelial cells were derived from healthy donors (*n* = 6) and COPD patients (*n* = 4) (50 % ≤ FEV1 < 80 % predicted). All the donors were off corticosteroid use in recent month. Airway epithelial cells were obtained by gently brushing the airway epithelium from the third- to fourth-order bronchi of healthy nonsmoking volunteers using flexible bronchoscopy as previously described [23]. Cells were collected and cultured in a T25 cell culture flask with BEGM (Lonza, Basel, Switzerland) in 5 % CO2 in a humidified chamber at 37 °C. The medium was changed after the first 12 h in culture to remove unattached cells and thereafter, every 2–3 days. Experiments were performed on BCs at passage 2 or 3.

### Cyto-immunofluorescence for the identification of BCs

After the first passage, BCs were seeded onto glass cover slips in a 24-well plate and incubated for 24 h. Cells were fixed in 4 % paraformaldehyde for 15 min and blocked with normal goat serum for 30 min at 37 °C. Cells were rinsed thoroughly with phosphate buffered saline (PBS) and incubated with primary antibody against Cytokeratin 5 (1:100; Abcam) and p63 (1:100; ZhongShan Golden Bridge Biotechnology) overnight at 4 °C. Slides were rinsed, incubated with TRITC conjugated anti-rabbit IgG (1:500; Beyotime, Beijing, China) and FITC conjugated anti-mouse IgG at room temperature for 30 min, counterstained with DAPI, and visualized with confocal fluorescence microscopy.

### Transfection

BCs were plated at a density of 10^5^ cells/well on a 24-well plate. After 24 h, cells were transfected with pcDNA3.1-TROP2 or siRNA sequence (5′-GCACGCUCAUCUAUUACCUTT-3′, 5′-AGGUAAUAGAUGAGCGUGCTT-3′) using Lipofectamine™ 2000 Transfection Reagent (Invitrogen, Carlsbad, CA, USA) according to the manufacturer’s instructions. Cells transfected with empty vector or scrambled siRNA were considered as negative control. At 48 h, cells were trypsinized, and experiments were performed.

### Quantitative real-time PCR

Total RNA was extracted from harvested cells using TRIzol reagent (Invitrogen, Carlsbad, CA, USA) according to the manufacturer’s instructions. First strand cDNA was synthesized from RNA (1 μg) using the ReverTra Ace® qPCR RT Kit (TOYOBO, Osaka City, Japan). Real-time PCR reactions (20 μL) were prepared using 2 μL of cDNA in SYBR® Green Real time PCR Master Mix (TOYOBO), and amplification was performed on an ABI PRISM 7900 HT Sequence Detection System (Applied Biosystems, Foster City, CA, USA). Relative quantification of mRNA was performed using the comparative C_T_ method where endogenous *GAPDH* was used as the normalization control. The primer sequences were as follows: *TROP2* forward primer, 5′-CGGCAGAACACGTCTCAGAAG-3′; reverse primer, 5′-CCTTGATGTCCCTCTCGAAGTAG-3′; *GAPDH* forward primer, 5′-GCACCGTCAAGGCTGAGAAC-3′; reverse primer, 5′-TGGTGAAGACGCCAGTGGA-3′. Results were obtained from three independent experiments performed in triplicate.

### Western blot analysis

Cell extracts were prepared by lysing cells in ice-cold RIPA buffer (20 mM sodium phosphate, 150 mM NaCl, pH 7.4, 1 NP-40, 0.1 SDS and 0.5 % deoxycholic acid) containing the protease inhibitor PMSF (Beyotime). Proteins (40 μg) were separated on 10 % SDS PAGE and transferred to PVDF membranes, and the blots were incubated with antibodies against human TROP2 (1 μg/ml), cyclin D1 (1:1000; Beyotime), E-cadherin (1:50000; Abcam), vimentin (1:500; Abcam) and GAPDH (1:2000; GoodhereBiotech Co., Hangzhou, China) as the protein loading control. Experiments were carried out in triplicate and repeated between three to five times.

### Cell proliferation and viability assay

Cell viability was assessed using a colorimetric assay, Cell Counting Kit-8 (CCK-8), according to the manufacturer’s instructions (Bestbio, Shanghai, China). Briefly, transfected and control cells were seeded onto 96-well plates at a density of 8000 cells/well and incubated in 5 % CO_2_ in a humidified chamber at 37 °C for 24, 48 or 72 h. At designated time intervals, CCK-8 solution (10 μL) was added to each well, and the optical density (O.D.) was measured at 450 nm in a microplate reader (Bio-Rad Model 680, Richmond, CA, USA) after a 3 h incubation at 37 °C. Experiments were repeated three times and six parallel holes were set in each experiment.

### Cell cycle analysis

Cells were transfected with pcDNA3.1-TROP2 or with siRNA sequence, harvested 48 h after transfection by trypsinization, and fixed in 75 % cold ethanol for 1 h at −20 °C. The cells were pelleted, rinsed with PBS, and incubated with 100 μL RNase A (100 mg/mL) and 400 μL propidium iodide for 30 min at 37 °C. Cell cycle analysis was performed on the FACSCalibur flow cytometer (Becton Dickinson, San Jose, CA, USA) at 488 nm. The relative ratios of the G1, S, and G2 phases were analyzed with ModFit LT 4.0 software. The assay was done in triplicate and repeated in three independent experiments.

### Wound repair

BCs were seeded onto 6-well plates, incubated overnight, and transfected with pcDNA3.1-TROP2 or with siRNA sequence. After reaching 90 % confluency, cell monolayers were scratched with a sterile pipette tip. Floating cells were removed with PBS and reseeded in BEGM medium. Images were taken at 0, 24 and 48 h to document the rate of migration of cells into the wound. Results were expressed as the ratio of the wound area detected at the designated time interval relative to the original scratch area. Repair was quantified using Image-Pro Plus software (Media Cybernetics, Rockville, MD, USA). Experiments were carried out in triplicate and repeated three times.

### Enzyme linked immunosorbent assay (ELISA)

Cells were transfected with pcDNA3.1-TROP2 or with siRNA sequence. The supernatants were harvested 48 h later, and the levels of IL-6, IL-8, and IL-1β were quantified in the supernatants using an ELISA kit according to the manufacturer’s instructions (RD Biosciences). The ELISA assay results were obtained from three independent experiments performed in triplicate.

### Statistical analysis

SPSS version 18.0 (SPSS Inc.; Chicago, IL, USA) was used to perform statistical analysis. All data were expressed as the mean ± the standard deviation (SD). The Kruskal–Wallis and Mann–Whitney U-tests were used for comparisons between patient groups, and correlation analyses were performed with Spearman rank correlation. The Student’s t test was used for analysis of in vitro experiments. *P* < 0.05 was considered statistically significant.

## Results

### Patient characteristics

Clinical characteristics and functional evaluation of the patient study groups are shown in Table 1. Importantly, the age range across the three patient groups was statistically similar, including that of the control nonsmoking group (*P* = 0.74). As patients with severe COPD could not tolerate thoracotomy surgery, they were not concerned in this study. As expected, the forced expiratory volume in first second percentage (FEV_1_%) of predicted and the FEV_1_/forced vital capacity (FVC) ratios were significantly reduced in smoking patients with COPD compared to healthy controls (*P* < 0.01 for both comparisons). The values in the FEV_1_% of predicted and the FEV_1_/FVC ratios were statistically similar between smokers without COPD and nonsmokers (*P* = 0.41 and *P* = 0.30, respectively). Although the smoking index in smokers with COPD seems to be a little higher than that in smokers without COPD, there was no statistical significance (*P* = 0.10).

**Table 1:** Patient clinical and functional characteristics

	Non-smokers	Smokers	Smokers with COPD
*n*	24	24	24
Sex (male/female)	20/4	19/5	19/5
Age (yr)	57.38 ± 9.36	55.58 ± 8.69	57.21 ± 8.28
Smoking index (pack-years)	0	34.79 ± 18.44	43.73 ± 16.00
FEV_1_ (% predicted)	98.49 ± 12.03	96.12.70 ± 8.01	63.24 ± 11.03^*#^
FEV_1_/FVC (%)	82.24 ± 6.02	80.54 ± 5.78	54.16 ± 8.38^*#^

*COPD*, chronic obstructive pulmonary disease, *FEV*_*1*_ forced expiratory volume in first second, *FVC* forced vital capacity. Data are expressed as the mean ± SD. ^*^ *P* < 0.01 *vs* non-smokers; ^#^ *P* < 0.01 *vs* smokers without COPD

### TROP2 expression is elevated in airway BCs in COPD lung tissue samples

**Fig. 1:**
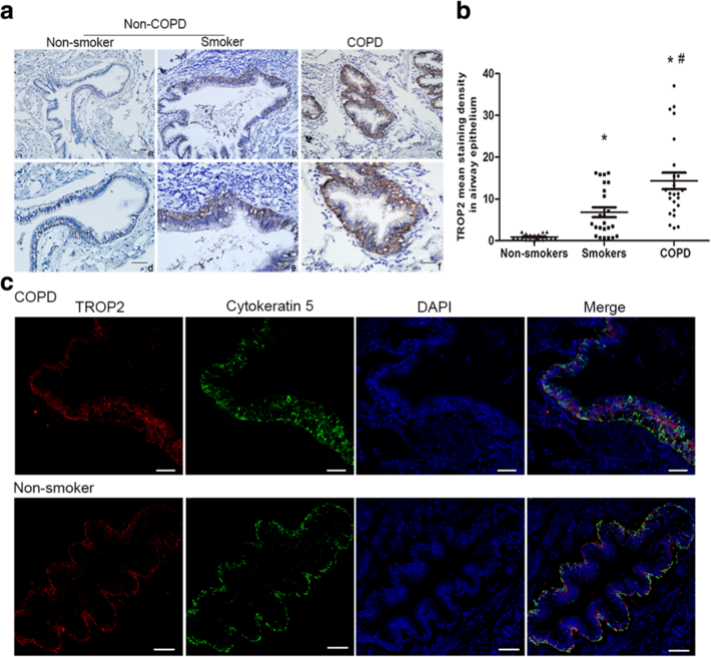
TROP2 expression is elevated in airway epithelium in lung tissue samples from COPD patients. **a** Immunohistochemistry staining for TROP2 performed on sections from non-smokers, smokers without COPD and smokers with COPD. Paired low and high magnification images are shown for each patient type to highlight localization of the staining. (*a*), (*b*), (*c*) × 100; (*d*), (*e*), (*f*) × 200; scale bars = 50 μm. **b** Quantification of TROP2 staining in sections from all patients (*n* = 72). **c** Immunofluorescent staining for TROP2 and cytokeratin 5 performed on sections from a smoker with COPD and a non-smoker. ^*^ *P* < 0.01 *vs* non-smokers; ^#^ *P* < 0.01 *vs* smokers without COPD

To study the potential role of TROP2 in the development of COPD, immunohistochemistry was used to compare TROP2 expression in lung tissue samples from smokers with COPD, smokers without COPD and nonsmokers (Fig. 1a). TROP2 was found to be expressed in airway epithelium of all patient samples. While only slight positivity of TROP2 was observed at the basolateral cytoplasmic membrane in bronchial epithelium of non-smokers, TROP2 was found to be highly expressed in airway epithelium of all smokers, especially in those patients with COPD (Fig. 1a). Quantitative analysis of the immunostaining revealed that TROP2 expression was significantly increased in COPD relative to nonsmoker and smoker controls as well as in smoker controls relative to nonsmokers (*P* < 0.01 for all; Fig. 1b).

One of the key findings of the immunostaining was that TROP2 was highly localized to cells along the basal membrane of the epithelium. To determine whether TROP2 was expressed specifically by airway BCs, immunofluorescent double staining was performed with cytokeratin 5, a molecular marker for BCs, and TROP2. Indeed, TROP2 staining was coincident with cytokeratin 5, indicating that expression of the protein originated in part from airway BCs (Fig. 1c).

### TROP2 expression correlates with the degree of airflow obstruction in COPD patients

**Fig. 2:**
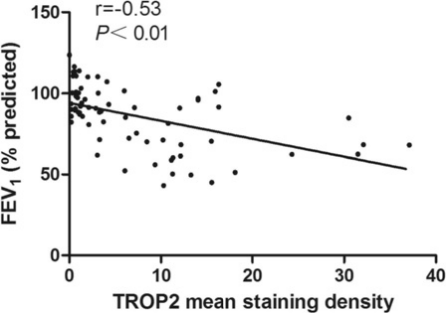
Correlation between the level of TROP2 protein expression based on immunostaining and FEV_1_%. Spearman rank correlation was used to examine the relationship between TROP2 expression and FEV_1_%. TROP2 expression is inversely correlated with FEV_1_% (r = −0.53, *P* < 0.01)

Molecular markers are increasingly playing a role in the pathologic and thus clinical diagnosis of disease. The severity of post-bronchodilator FEV_1_% of predicted is recommended for the assessment of COPD severity according to GOLD. Therefore, correlation of the level of TROP2 expression with FEV_1_% was examined in our patient cohort. The analysis revealed a significant inverse correlation between FEV_1_% and TROP2 expression, i.e. increases in TROP2 were coordinate with a decreased FEV_1_% of predicted (r = −0.53, *P* < 0.01; Fig. 2).

### Increased TROP2 expression correlates with a high airway proliferative index in COPD tissue samples

**Fig. 3:**
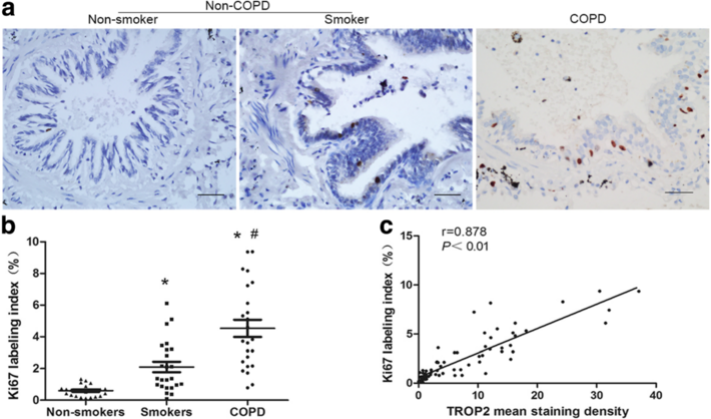
Expression of TROP2 correlates with proliferative index of airway epithelium based on Ki67. **a** Immunohistochemistry detecting Ki67 expression in sections of paraffin embedded lung tissues from patients as indicated. Positive staining for Ki67 correlates with the brown staining from DAB. Original × 200; scale bars = 50 μm. **b** Quantitation of immunostaining for Ki67 in patient samples (*n* = 72) as indicated. The density of Ki67 positive cells was significantly higher in hyperplasic epithelium of COPD patients than in normal airway epithelium (*P* < 0.01). **c** Correlation of the level of TROP2 protein expression with Ki67 in COPD patient samples as determined by immunohistochemistry. Results indicated a positive correlation between increased TROP2 and Ki67 positive cells. ^*^ *P* < 0.01 *vs* non-smokers; ^#^ *P* < 0.01 *vs* smokers without COPD

Airway epithelial hyperplasia is one of the key pathological features of COPD. To explore a possible association of TROP2 with airway hyperplasia in COPD, the localization of the proliferation marker Ki67 and the percentage of Ki67 positive cells were compared to immunostaining for TROP2 in patient samples. The density of Ki67 positive cells was significantly higher in hyperplasic epithelium of COPD patients than that in normal airway epithelium, thus paralleling the staining pattern of TROP2 (*P* < 0.01; Fig. 3a and b). Analysis of immunostaining for the two proteins revealed a statistically significant correlation between increased TROP2 expression and Ki67 positive cells in COPD lung tissue samples (*r* = 0.878, *P* < 0.01; Fig. 3c).

### TROP2 promotes proliferation and wound closure in BCs in vitro

**Fig. 4:**
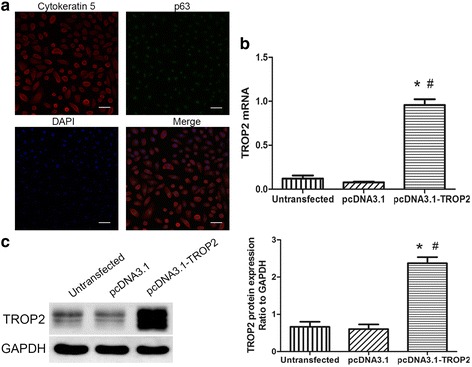
BCs transfected with pcDNA3.1-TROP2 express TROP2 RNA and protein. **a** Immunofluorescent staining of cultures for the stem cell markers p63 and cytokeratin 5 (red) to identify BCs isolated from airway epithelium of healthy volunteers. Cytokeratin 5 is expressed in the cytoplasm while p63 is expressed in the nuclei. DAPI highlights nuclei of viable cells. Original × 200; scale bars = 50 μm. **b** mRNA expression of *TROP2* in pcDNA3.1-TROP2-BCs. Relative levels of *TROP2* expression to *GAPDH* in the indicated cell types determined using the δC_T_ in transfected *vs* control cells. **c** Protein expression levels of TROP2 in pcDNA3.1-TROP2-BCs. Western blot prepared with cell lysates (40 μg) from transfected and control cells and incubated with the antibodies indicated. Protein levels were quantitated by chemiluminescence with GAPDH used as the control for protein loading. ^*^ *P* < 0.01 *vs* untransfected cells; ^#^ *P* < 0.01 *vs* pcDNA3.1-BCs

Given the close association between elevated TROP2 expression and airway epithelial hyperplasia in COPD patient lung tissues, TROP2 might promote proliferation of BCs in COPD patients. In order to begin to elucidate the functional consequences of increased TROP2 expression, an in vitro system was exploited. BCs were first isolated from airway epithelium of healthy volunteers, cultured, and characterized using immunofluorescent staining for markers of airway BCs, cytokeratin 5 and p63 (Fig. 4a). BCs were subsequently transfected with an expression vector for TROP2, pcDNA3.1-TROP2. At 48 h post-transfection, mRNA and protein levels of TROP2 were increased by ~2.5 fold in BCs, as assessed by real-time PCR and Western blots analysis (Fig. 4b and c).

**Fig. 5:**
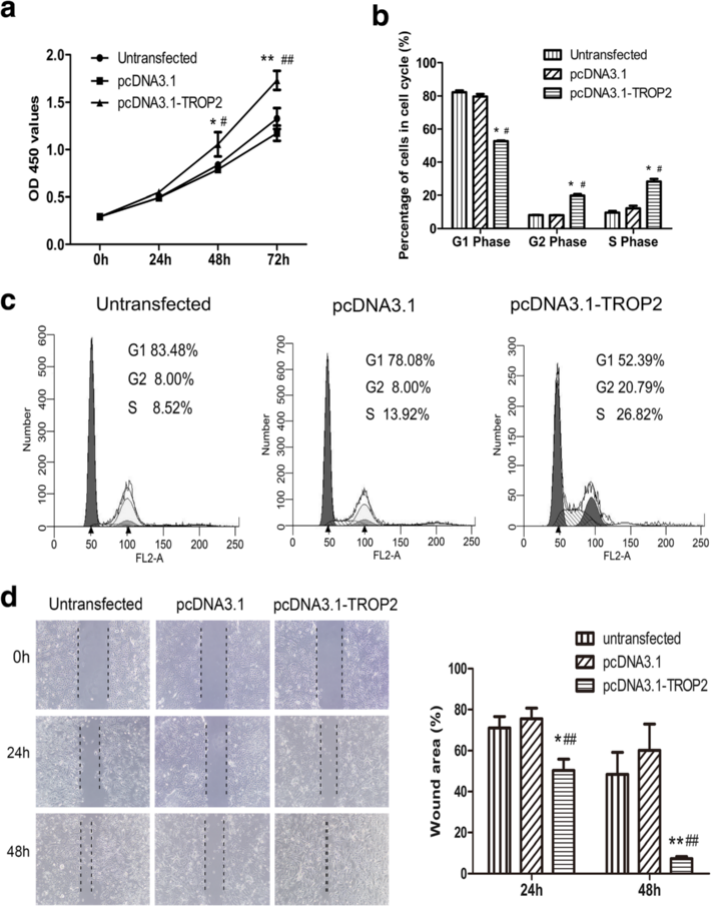
TROP2 promotes BC proliferation and wound closure in vitro. **a** The O.D. 450 results from the CCK-8 viability assay are plotted as a function of time for pcDNA3.1-TROP2-BCs and control cells. Cell viability in the pcDNA3.1-TROP2-BCs was significantly improved (*P* < 0.05). **b**, **c** Cell cycle analysis of pcDNA3.1-TROP2-BCs and control cells. Distribution of cells in G1, S and G2/M phases as determined by flow cytometry are represented graphically in a bar graph and in the standard flow diagram generated in the ModFit LT 4.0 software. **d** Images of wound healing assays and quantitation of results performed on pcDNA3.1-TROP2-BCs and control cell types. (*P* < 0.01). ^*^ *P* < 0.05 *vs* untransfected cells; ^#^ *P* < 0.05 *vs* pcDNA3.1-BCs; ^**^ *P* < 0.01 *vs* untransfected cells; ^##^ *P* < 0.01 *vs* pcDNA3.1-BCs

Two assays were performed to assess the possible function of TROP2 in promoting the proliferation of BCs: the CCK-8 assay to measure cell viability which is directly proportional to increases in cell number, and flow cytometry to assess cell cycle progression. In the CCK-8 assay, O.D. 450 values were increased in pcDNA3.1-TROP2-BCs, indicating that cell viability was significantly improved in these cells relative to controls (*P* < 0.05; Fig. 5a). When coupled with cell cycle analysis, the increases in O.D. 450 associated with TROP2 expression were found to be due to changes in cell cycle kinetics. The percentage of cells in S and G2/M was significantly increased in pcDNA3.1-TROP2-BCs relative to control cells (*P* < 0.05; Fig. 5b and c).

Enhanced proliferation in COPD is a consequence of perpetual wound healing or tissue repair occurring in COPD tissues of patients. The function of increased TROP2 expression in COPD was further investigated in BCs in vitro in a wound healing assay. At 24 and 48 h after wounding, pcDNA3.1-TROP2-BCs displayed an increased capacity for wound closure relative to control cells (*P* < 0.01; Fig. 5d). The increased wound healing response upon TROP2 overexpression can be due to increased proliferative responses.

### Down-regulated TROP2 decreases proliferation and wound closure in COPD-derived BCs

**Fig. 6:**
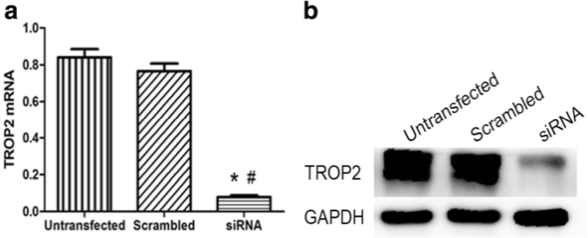
Down-regulation of TROP2 by siRNA in COPD-derived BCs. **a** mRNA expression of *TROP2* in siRNA-TROP2-BCs. **b** Protein expression levels of TROP2 in siRNA-TROP2-BCs. ^*^ *P* < 0.01 *vs* untransfected cells; ^#^ *P* < 0.01 *vs* Scrambled-BCs

In order to show the relevance of endogenous TROP2 expression, TROP2 was down-regulated by siRNA in COPD-derived BCs. At 48 h post-transfection, the result of real-time PCR and Western blots analysis showed that mRNA and protein levels of TROP2 were decreased by ~4.5 fold in BCs (Fig. 6a and b).

**Fig. 7:**
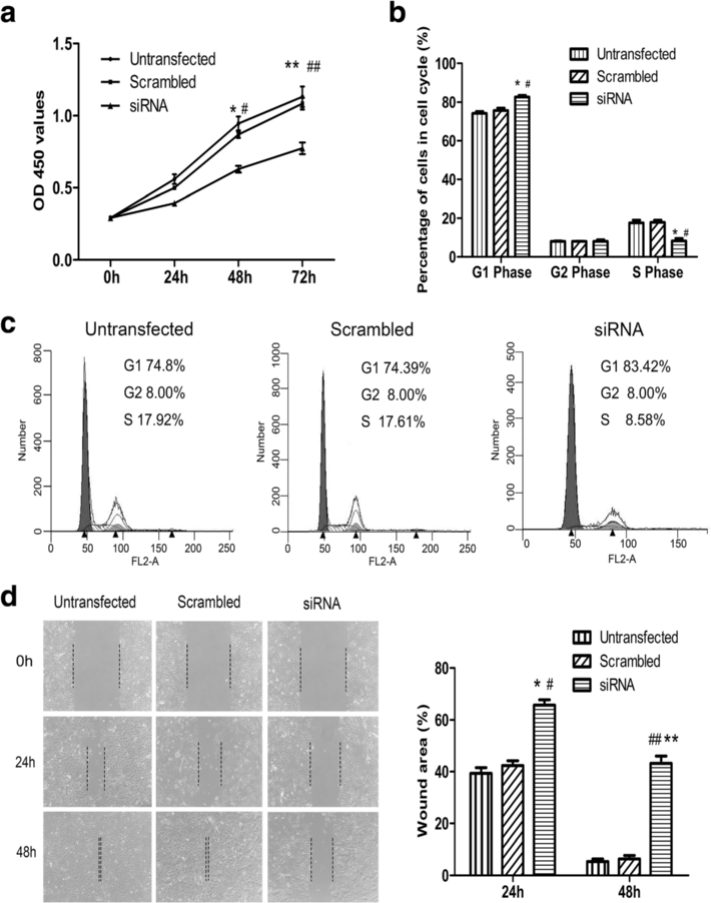
Down-regulated TROP2 decreases proliferation and wound closure in COPD-derived BCs. **a** Cell viability in the siRNA-TROP2-BCs was significantly inhibited (*P* < 0.05). **b**, **c** Cell cycle analysis of siRNA-TROP2-BCs and control cells. **d** Images of wound healing assays and quantitation of results performed on siRNA-TROP2-BCs and control cell types. ^*^ *P* < 0.05 *vs* untransfected cells; ^#^ *P* < 0.05 *vs* siRNA-BCs; ^**^ *P* < 0.01 *vs* untransfected cells; ^##^ *P* < 0.01 *vs* siRNA-BCs

The CCK-8 assay showed that cell viability of siRNA-TROP2-BCs was significantly impaired compared with controls (*P* < 0.05; Fig. 7a). Accordingly, the percentage of cells in S was significantly decreased in siRNA-TROP2-BCs relative to control cells (*P* < 0.05; Fig. 7b and c). The wound healing assay revealed that, at 24 and 48 h after wounding, siRNA-TROP2- BCs exhibited a weakened capacity for wound closure relative to control cells (*P* < 0.05; Fig. 7d).

### Activation of ERK/MAPK signaling stimulates BC proliferation

**Fig. 8:**
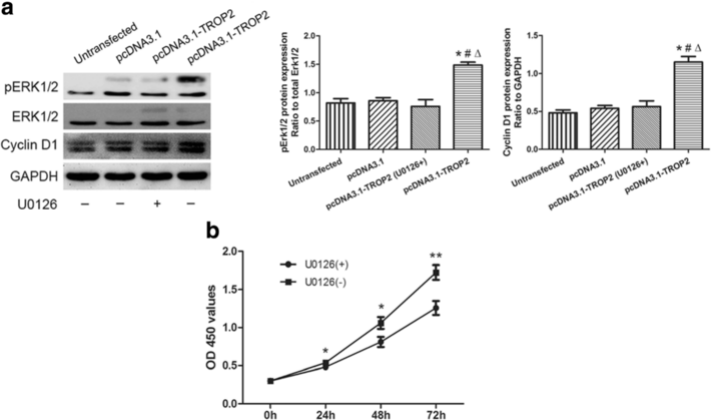
Activation of the ERK/MAPK signaling pathway enhances proliferation of BCs. **a** Western blot analysis was performed on cell lysates (40 μg) prepared from transfected (pcDNA3.1 and pcDNA3.2-TROP2) and control cells 48 h after transfection and inhibition of the ERK/MAPK pathway with U0126 as indicated (+/−). Blots were incubated with the antibodies indicated. Protein levels were visualized with chemiluminescence, and GAPDH was used as the control for protein loading. **b** O.D. 450 nm of CCK-8 was plotted as a function of time to assess viability/proliferation of pcDNA3.1-TROP2-BCs treated with the ERK/MAPK pathway inhibitor U0126. ^*^ *P* < 0.05; ^**^ *P* < 0.01

Recent studies have linked TROP2 to the MAPK signaling pathway in the regulation of the cell cycle and cell proliferation. Western blot analysis was performed to determine whether phosphorylation of ERK1/2 might also contribute to the altered cell proliferation mediated by TROP2 in airway BCs in vitro. At 48 h after transfection, protein levels of pERK1/2 were up-regulated in pcDNA3.1-TROP2-BCs (Fig. 8a). Expression of cyclin D1, a downstream molecule in the ERK1/2 MAPK signaling pathway and a positive regulator of G1 to S phase transition, was also increased significantly. A second approach to investigate the intersection of these pathways was to treat pcDNA3.1-TROP2-BCs with a specific inhibitor of the MAPK pathway, U0126 (20 μM; Beyotime). Treatment with U0126 blocked the increase in pERK1/2 and inhibited TROP2 mediated BC proliferation assessed by CCK-8 assay (Fig. 8b), indicating that pERK1/2 might be involved in the signaling pathway.

### TROP2 induces an epithelial-mesenchymal transition (EMT)-like phenotype in proliferating airway BCs

**Fig. 9:**
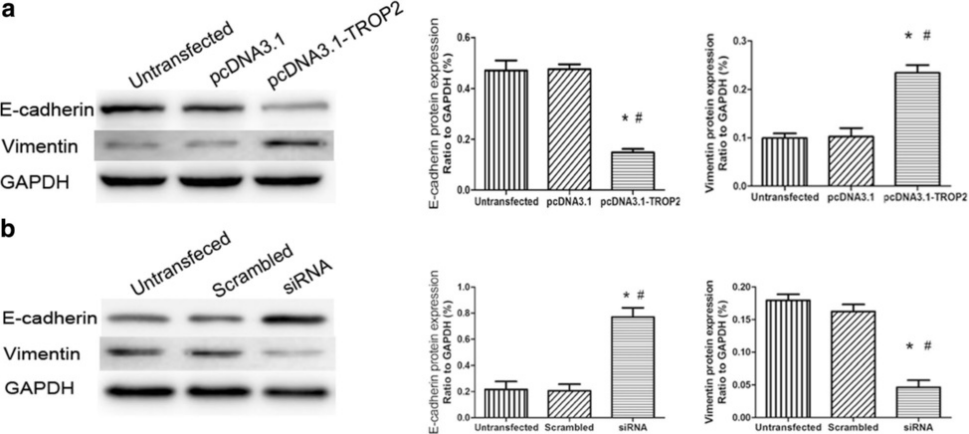
The effect of TROP2 expression on EMT-like changes in BCs. Western blot analysis was performed on cell lysates (40 μg) prepared from cells transfected with the pcDNA3.1 or the expression vector pcDNA3.1-TROP2 and control BCs. Blots were incubated with the antibodies as indicated with GAPDH used as the control for protein loading. Protein levels indicated were quantitated based on chemiluminescence. **a** The protein levels of the epithelial marker E-cadherin was significantly decreased in pcDNA3.1-TROP2-BCs (*P* < 0.01) whereas the expression of the mesenchymal marker vimentin was increased compared to control cells (*P* < 0.01). ^*^ *P* < 0.01 *vs* untransfected cells; ^#^ *P* < 0.01 *vs* pcDNA3.1-BCs. **b** Down-regulation of TROP2 significantly increased E-cadherin expression and decreased vimentin expression in COPD-derived BCs (*P* < 0.01). ^*^ *P* < 0.01 *vs* untransfected cells; ^#^ *P* < 0.01 *vs* Scrambled-BCs

EMT is an important pathological feature of airway remodeling which occurs in COPD, and it may be related to altered fibrotic activity taking place in the sub-epithelial tissue. TROP2 has in fact been reported to be involved in inducing EMT in some epithelial cell types, and as such, is a potential candidate gene involved in the phenomenon in airway BCs. Western blot analysis was used to determine protein levels of specific markers typically altered in EMT in pcDNA3.1-TROP2-BCs and siRNA-TROP2-BCs in culture. Protein levels of the epithelial marker E-cadherin was significantly decreased in pcDNA3.1-TROP2-BCs relative to control cells (*P* < 0.01; Fig. 9a) whereas the expression of the mesenchymal marker vimentin was increased (~2.5 fold; *P* < 0.01; Fig. 9a). In contrast, down-regulation of TROP2 significantly increased E-cadherin expression and decreased vimentin expression in COPD-derived BCs (*P* < 0.01; Fig. 9b)

.

### Secretion of proinflammatory cytokines is increased in BCs overexpressing TROP2

**Fig. 10:**
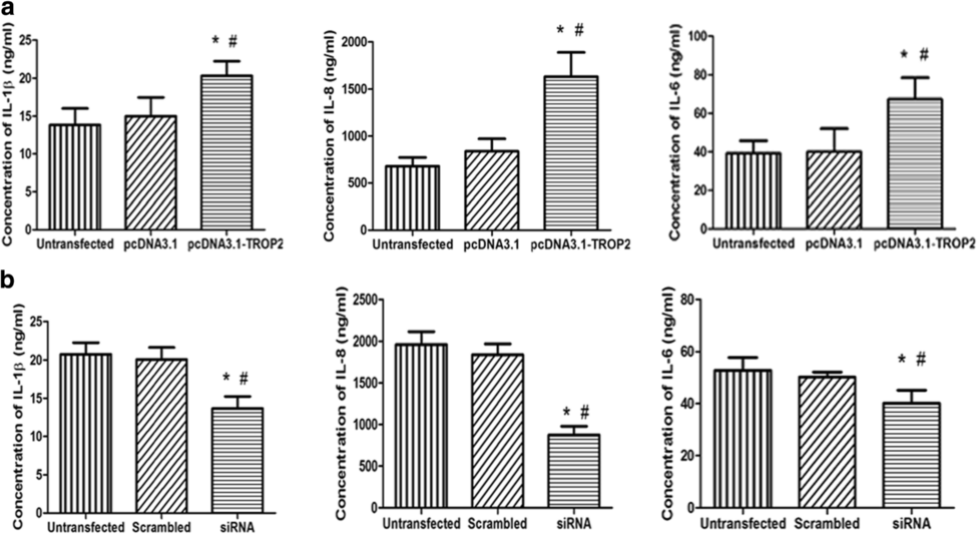
The influence of TROP2 expression on secretion of proinflammatory cytokines in BCs. BCs were transfected with pcDNA3.1-TROP2 or siRNA sequence. The concentrations of IL-1β IL-6, and IL-8 in supernatants of transfected and control cells were analyzed by ELISA and compared. The results represent the mean ± SD of 3 measurements. **a** Secretion of IL-1β IL-6, and IL-8 was increased in BCs overexpressing TROP2. *P* < 0.05 for all factors. ^*^ *P* < 0.05 *vs* untransfected cells; ^#^ *P* < 0.05 *vs* pcDNA3.1-BCs. **b** Secretion of IL-1β IL-6, and IL-8 was significantly decreased in TROP2-downregulated BCs (*P* < 0.05). ^*^ *P* < 0.01 *vs* untransfected cells; ^#^ *P* < 0.01 *vs* Scrambled-BCs

An important factor contributing to significant changes in tissue architecture is the secretion of proinflammatory cytokines into the microenvironment. To determine whether increased expression of TROP2 influenced secretion of inflammatory factors from proliferating BCs, the levels of inflammatory cytokines IL-6, IL-8, and IL-1β were evaluated in supernatants from transfected BCs in vitro. The levels of all three factors were increased in the supernatants of pcDNA3.1-TROP2-BCs relative to supernatants of control cells (*P* < 0.05 for all factors; Fig. 10a). On contrary, after the TROP2 expression was down-regulated by siRNA in COPD-derived BCs, the levels of IL-6, IL-8, and IL-1β in supernatants were significantly decreased (*P* < 0.05 for all factors; Fig. 10b).

## Discussion

Airway BCs play crucial roles in epithelial regeneration and homeostasis following injury [24]. Disregulated BC function has been demonstrated to be an early event in the pathogenesis of COPD, based in part on changes in BC transcription in smokers and as the smoker transitions into clinically defined COPD [25–27]. BC hyperplasia has been a histologic abnormality associated with smoking [28], although the reasons remain obscure. Because of its association with proliferation and progenitor cells in mouse and human, TROP2 was the focus of our study as a potential regulator of the biological behavior of airway BCs in COPD.

Our results demonstrated that TROP2 expression was significantly increased in vivo in airway BCs of COPD patients, and revealed a significant correlation between TROP2 expression and the degree of airway epithelium hyperplasia. In vitro, TROP2 promoted BC proliferation and self-renewal mediated possibly through the ERK signaling pathway. Finally, TROP2 induced an EMT-like phenotype and release of proinflammatory cytokines in proliferating BCs. These results indicate that increased TROP2 protein in BCs might play a critical role in airway remodeling which is a key pathological feature of COPD in patients.

Regulation of TROP2 gene expression has been detected by DNA microarray analysis in cancer cells [29]. Whereas little is known about the mechanisms that could be responsible for the increased expression of TROP2 in COPD epithelium. In our study, staining intensity was generally greater in smokers regardless of COPD status than in nonsmokers, indicating that smoking alone was a risk factor up-regulating TROP2 expression. Smoking was not however the single contributing factor, as higher TROP2 expression was specifically observed in patients with COPD. One explanation for this result might be an individual genetic susceptibility to COPD. Further research is necessary to investigate the exact regulatory mechanism of TROP2 expression in airway BCs. Further, the relationship between TROP2 and cell proliferation due to the expression of the proliferation-related nuclear protein Ki67 was established in this study. The significant correlation between TROP2 and Ki67 expression supports the hypothesis that overexpressed TROP2 is closely related to the hyperproliferative state of airway epithelium in COPD.

The results from immunohistochemistry were further supported by experiments performed in an in vitro system, which revealed an enssential role that TROP2 played in airway BC proliferation. It has been reported that U0126 (20 μM) is a more highly selective inhibitor of MEK1/2, although it might have a very weak inhibitory effect on PKC, Cdk2 and Cdk4 [30]. Therefore, our result suggested that pERK1/2 MAPK signal pathway might be involved in the process of TROP2-mediate BC proliferation. The mechanism of TROP2-induced activation of pERK1/2 in BCs is unclear. It has been reported that overexpressed TROP2 could increase the intracellular Ca^2+^ level, which was capable of activating the ERK MAPK pathways through calmodulin-dependent protein kinases [13]. TROP2 regulated cell self-renewal and proliferation through a mechanism of regulated intramembrane proteolysis was found in prostate stem cells [14].

BC hyperplasia is a typical airway epithelial remodeling phenotype in COPD lungs. The proliferation of BCs can act as a benign protective cellular reaction to injury in the airways of normal individuals, but it may also be the cause of pathological changes occurring at an early stage in COPD. BC hyperplasia in small airways could lead to airway wall thickening, contributing to small airway narrowing and airflow limitation [31]. Furthermore, the increased number of BCs also gives rise to squamous metaplasia in central airways, which is also an early airway pathological change observed in COPD patients [32, 33].

BCs acquired EMT-like features during TROP2 mediated proliferation, which was manifested by the decreased expression of epithelial cell markers cytokeratin 5 and E-cadherin and the acquisition of the mesenchymal cell marker vimentin. Classical EMT refers to the epithelial cells closely connected to the basement membrane which lose polarity and acquire a mesenchymal cell phenotype [34]. The EMT process is present in bronchial epithelial cells of patients with COPD and has a role in airway remodeling [35, 36]. Our findings indicate that BCs may be one of the cell types undergoing EMT. Consistent with this result, is that BCs have been reported to undergo EMT-like changes in an acute lung injury model of BC differentiation which was mediated by EGF. Airway epithelium generated from the EGF-exposed BCs also exhibited decreased barrier integrity [37]. In addition, Jonsdottir *et al*. recently reported that some p63-positive BCs are prone to phenotypic changes and could act as EMT progenitors in idiopathic pulmonary fibrosis [38].

Exposure to repetitive inflammatory stimuli leads to exaggerated and persistent activation of the repair process and is one of the primary causes of airway remodeling characteristic of COPD [39, 40]. In our study, secretion of proinflammatory cytokines IL-6, IL-8, and IL-1β was increased in TROP2 mediated proliferation of BCs. Elevated levels of these proinflammatory factors have been confirmed in the airways of COPD patients. These cytokines serve as effective chemokines, leading to the infiltration of neutrophils and macrophages and the expansion of persistent inflammatory injury to airway epithelium [41]. BCs are not just cellular targets of the process of inflammation; BCs also release chemokines and cytokines, thereby initiating and orchestrating immune and inflammatory responses. These data support the concept that the pathological change secondary to altered BC proliferation leads to abnormal innate immunity, potentially contributing to the pathologic cycle of chronic airway inflammation and remodeling in COPD.

One limitation of this study is that all the lung tissue samples were collected from patients with mild to moderate COPD, and severe COPD patients incapable of undergoing surgery operation were not included. The situation in severe COPD may be very different from the relative early stage, in which the repairing ability of BCs may be relative conserved. As Perotin et al. reported that delayed rather than enhanced epithelial wound repair was found in severe COPD [42]. Actually, we agree with this idea. The explanation for this observation may be that the renewal and regeneration capacity of BCs could diminish due to excessive stem cell hyperproliferation and eventual replicative senescence [43, 44]. And we also observed a so-called aging of pcDNA3.1-TROP2-BCs in culture in cells passaged > 5 generations. This finding parallels the in vivo situation of COPD: an initial promotion of BC proliferation followed by senescence or death after extended time in the repair state. In addition, the mechanism involved in TROP2 inducing EMT-like changes and enhanced proinflammatory cytokines secretion has not been deeply researched yet. pERK1/2 MAPK signal pathway was considered in the study. After pre-treated with U0126, neither EMT makers nor proinflammatory cytokine concentrations underwent significant changes in pcDNA3.1-TROP2-BCs (data not shown), suggesting that pERK1/2 MAPK pathway may not participate in the process. It is necessary to carry on further researches in the subsequent studies.

## Conclusions

In summary, the data presented in this study identify a potential role for TROP2 overexpression in the pathogenesis of COPD. TROP2 expression was particularly enriched in airway BCs of patients with COPD, and in vitro, TROP2 overexpression induced changes in BCs that parallel airway remodeling in COPD. TROP2 might therefore play a critical role in early airway repair abnormalities and remodeling in COPD patients. Further insight into the function of TROP2 in BCs will provide potential strategies for future therapy of COPD.

## Abbreviations

BCs: Basal cells
COPD: Chronic obstructive pulmonary disease
EMT: Epithelial-mesenchymal transition
ERK: Extracellular regulated protein kinase
FEV_1_: Forced expiratory volume in first second
FVC: Forced vital capacity
GOLD: Global Initiative for Chronic Obstructive Lung Disease
TROP2: Trophoblast cell surface antigen 2
